# Quantitative analysis of islet prohormone convertase 1/3 expression in human pancreas donors with diabetes

**DOI:** 10.1007/s00125-024-06275-5

**Published:** 2024-10-15

**Authors:** Paola S. Apaolaza, Yi-Chun Chen, Kavi Grewal, Yannik Lurz, Severin Boulassel, C. Bruce Verchere, Teresa Rodriguez-Calvo

**Affiliations:** 1 Institute of Diabetes Research, Helmholtz Zentrum München, German Research Center for Environmental Health, Munich-Neuherberg, Germany; 2 Department of Surgery, University of British Columbia & BC Children’s Hospital Research Institute, Vancouver, BC Canada; 3 Technical University of Munich, Munich, Germany; 4 Department of Pathology and Laboratory Medicine, University of British Columbia & BC Children’s Hospital Research Institute, Vancouver, BC Canada; 5 Centre for Molecular Medicine and Therapeutics, University of British Columbia, Vancouver, BC Canada

**Keywords:** Beta cell dysfunction, Prohormone convertase 1/3, Proinsulin, Quantitative image analysis

## Abstract

**Aims/hypothesis:**

Islet prohormone-processing enzymes convert peptide hormone precursors to mature hormones. Defective beta cell prohormone processing and the release of incompletely processed peptide hormones are observed prior to the onset of diabetes, yet molecular mechanisms underlying impaired prohormone processing during the development of diabetes remains largely unknown. Previous studies have shown that prohormone convertase 1/3 (PC1/3) protein and mRNA expression levels are reduced in whole islets from donors with type 1 diabetes, although whether PC1/3-mediated prohormone processing in alpha and beta cells is disrupted in type 1 diabetes remained to be explored. Herein, we aimed to analyse the expression of PC1/3 in islets from non-diabetic donors, autoantibody-positive donors and donors diagnosed with type 1 diabetes or type 2 diabetes.

**Methods:**

Immunostaining and high-dimensional image analysis were performed on pancreatic sections from a cross-sectional cohort of 54 donors obtained from the Network for Pancreatic Organ Donors with Diabetes (nPOD) repository, to evaluate PC1/3 expression patterns in islet alpha, beta and delta cells at different stages of diabetes.

**Results:**

Alpha and beta cell morphology were altered in donors with type 1 diabetes, including decreased alpha and beta cell size. As expected, the insulin-positive and PC1/3-positive areas in the islets were both reduced, and this was accompanied by a reduced percentage of PC1/3-positive and insulin-positive/PC1/3-positive cells in islets. PC1/3 and insulin co-localisation was also reduced. The glucagon-positive area, as well as the percentage of glucagon-positive and glucagon-positive/PC1/3-positive cells in islets, was increased. PC1/3 and glucagon co-localisation was also increased in donors with type 1 diabetes. The somatostatin-positive cell area and somatostatin staining intensity were elevated in islets from donors with recent-onset type 1 diabetes.

**Conclusions/interpretation:**

Our high-resolution histomorphological analysis of human pancreatic islets from donors with and without diabetes has uncovered details of the cellular origin of islet prohormone peptide processing defects. Reduced beta cell PC1/3 and increased alpha cell PC1/3 in islets from donors with type 1 diabetes pinpointed the functional deterioration of beta cells and the concomitant potential increase in PC1/3 usage for prohormone processing in alpha cells during the pathogenesis of type 1 diabetes. Our finding of PC1/3 loss in beta cells may inform the discovery of new prohormone biomarkers as indicators of beta cell dysfunction, and the finding of elevated PC1/3 expression in alpha cells may encourage the design of therapeutic targets via leveraging alpha cell adaptation in diabetes.

**Graphical Abstract:**

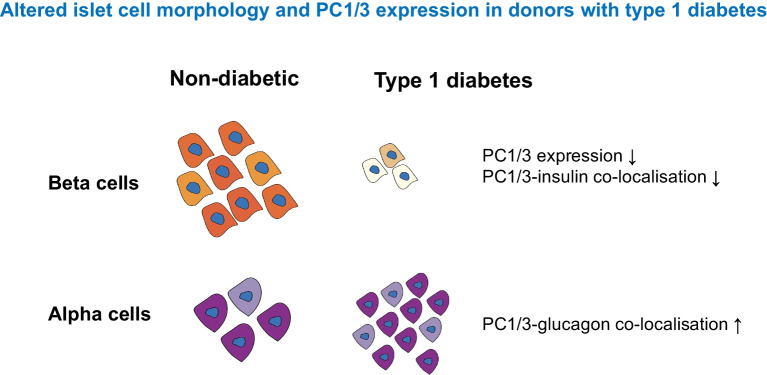

**Supplementary Information:**

The online version contains peer-reviewed but unedited supplementary material available at 10.1007/s00125-024-06275-5.


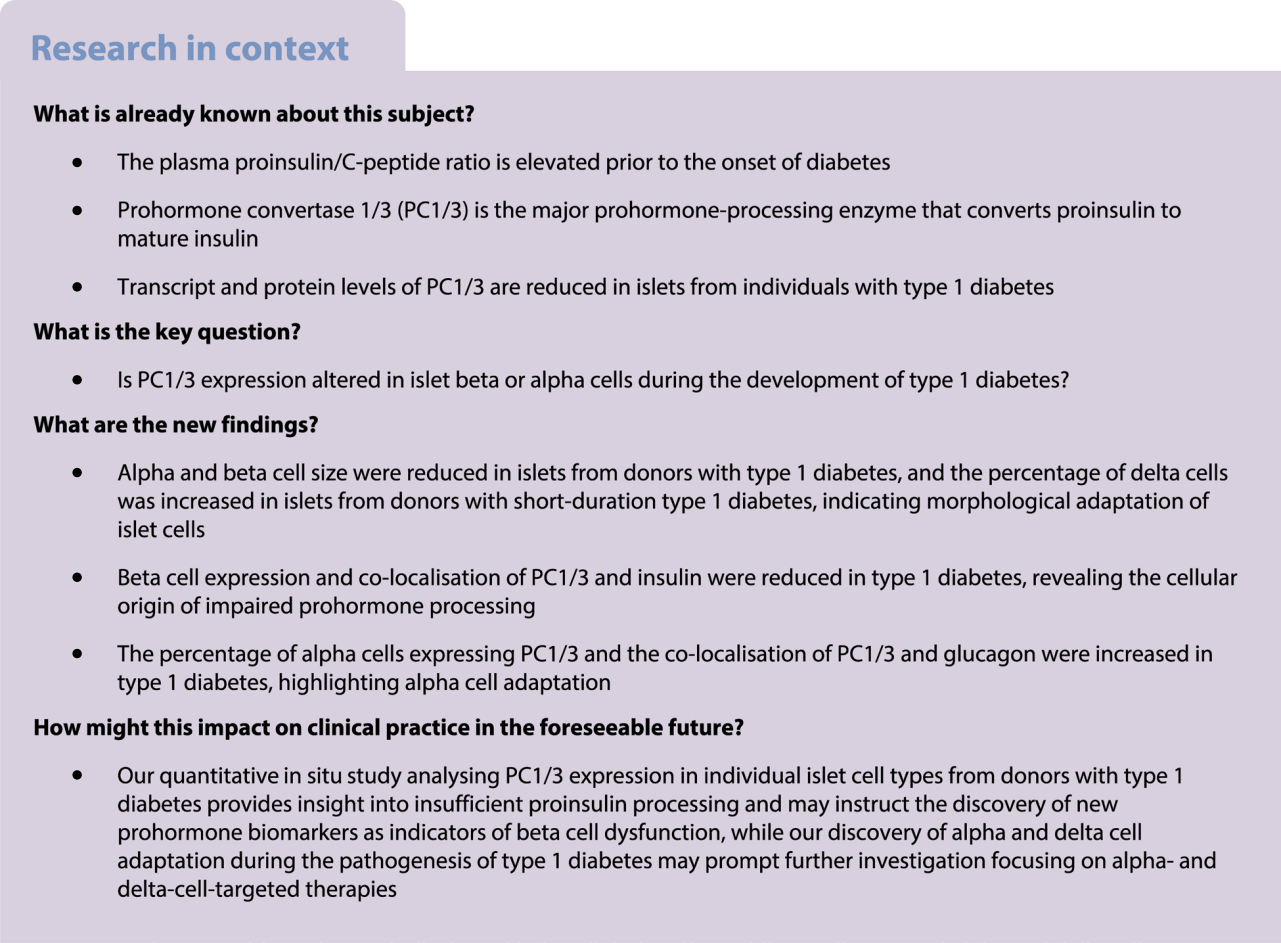


## Introduction

Type 1 diabetes is an autoimmune disease that culminates with the immune-mediated loss of pancreatic beta cells [1]. Histopathological observations from biopsy, autopsy and donor pancreases have unveiled the progressive loss of beta cells during the development of type 1 diabetes [2]. Contrary to previous beliefs, beta cell mass is highly variable among individuals and is not entirely correlated with the degree of insulitis, autoantibody status or duration of disease [3]. Decreased beta cell function, on the other hand, is associated with the progression to type 1 diabetes [4] and can be measured via circulating indicators such as beta cell peptide hormones (i.e. proinsulin and C-peptide) [5–7].

Peptide hormones are made as larger precursors. Via enzyme-mediated processing, propeptide hormones are converted to mature hormones. For example, proinsulin is processed by prohormone convertase 1/3 (PC1/3), prohormone convertase 2 (PC2) and carboxypeptidase E (CPE) to yield mature insulin and its byproduct C-peptide. Similarly, pro-islet amyloid polypeptide (proIAPP), another abundantly expressed beta cell peptide hormone, is processed by PC1/3, PC2, CPE and peptidyl-α-amidating monooxygenase (PAM) to form mature amidated islet amyloid polypeptide (IAPP). Defects in prohormone processing lead to elevated circulating levels of prohormones and reduced mature hormones, which can be measured via commercial or in-house developed immunoassays [8]. Despite being a promising indicator of beta cell dysfunction in both type 1 diabetes and type 2 diabetes, the root cause of impaired prohormone processing remains elusive. PC1/3 protein and mRNA levels are reduced in the pancreas and insulin-containing islets from donors with type 1 diabetes [9–11], yet PC1/3 protein levels in individual beta cells have not been quantitatively analysed. Elevated peptide hormone precursors may create protein-folding stress in beta cells or act as neoantigens that contribute to immune activation, facilitating the progression to type 1 diabetes [12]. On the other hand, PC1/3 mis-expression in islet alpha cells may result in alternative processing of proglucagon to glucagon-like peptide 1 (GLP-1) [13–15], which potentiates glucose-stimulated insulin secretion and promotes beta cell survival [16]. PC1/3 is also highly expressed in delta cells, yet its role in secretory granule trafficking and peptide processing remains elusive [17, 18]. A better understanding of PC1/3 expression in islet cells from autoantibody-positive (AAb^+^) donors and donors with diabetes may facilitate the identification of additional prohormone biomarkers of beta cell dysfunction or inform the design of therapeutic targets via leveraging alpha and delta cell adaptation in diabetes.

To this end, PC1/3 protein expression in individual alpha, beta, and delta cells from AAb^+^ donors and donors with recent-onset or short duration type 1 diabetes (<5 years), long-duration type 1 diabetes (>15 years), type 2 diabetes and non-diabetic donors were evaluated via multiplex immunostaining, confocal imaging and high-dimensional image analyses, including islet cell morphology, PC1/3 expression and co-localisation of insulin and glucagon at the single-cell level, as well as glucagon- and somatostatin-positive areas in the islets.

## Methods

### Pancreas donor characteristics

Formalin-fixed paraffin-embedded sections from the tail of the pancreas of organ donors were obtained from the Network for Pancreatic Organ Donors with Diabetes (nPOD) [19]. Pancreatic sections from a total of 54 donors were analysed: eight with islet autoantibodies (three double-autoantibody and five single-autoantibody donors; age [mean ± SEM] 20.8 ± 1.6 years); ten with short-duration type 1 diabetes (age 20.3 ± 1.7 years, time since diagnosis 3.2 ± 0.5 years); ten with long-duration type 1 diabetes (age 41.8 ± 4.5 years, time since diagnosis 24.3 ± 4.0 years); six with type 2 diabetes (age 42.6 ± 3.9 years, time since diagnosis 30.6 ± 5.3 years); ten non-diabetic young adults (age 20.3 ± 1.4 years); and ten non-diabetic adult (age 42.9 ± 1.9 years). Additional donor characteristics including gender, BMI, age at onset, C-peptide and pancreas weight are listed in Table 1. All pancreatic samples from donors with short- or long-duration type 1 diabetes retained insulin-containing islets and had no reported pancreatic malignancy. The study was approved by the University of British Columbia Institutional Review Board.

**Table 1:** Donor characteristics

Diabetes status	nPOD case ID	Age, years	Gender, *n* male / *n* female	BMI, kg/m^2^	Diabetes duration, years	Age at onset, years	C-peptide, (pmol/l)	Pancreas weight, g
ND (young adults)	6387, 6338, 6271, 6174, 6172, 6179, 6238, 6417, 6131, 6229	20.3 ± 1.4	6/4	23.6 ± 1.3			4.7 ± 1.0	75.7 ± 6.89
AAb^+^	6123, 6147, 6314, 6347, 6400 (single AAb^+^) 6197, 6429, 6450 (double AAb^+^)	20.8 ± 1.6	5/3	23.4 ± 1.6			4.8 ± 2.1	62.7 ± 4.39
Recent-onset T1D (<5 years)	6247, 6469, 6371, 6396, 6198, 6084, 6087, 6212, 6306, 6441	20.3 ± 1.7	6/4	23.2 ± 1.3	3.2 ± 0.5	17.0 ± 1.8	0.1 ± 0.1	45.7 ± 6.24
Long-duration T1D (>15 years)	6285, 6208, 6321, 6077, 6231, 6328, 6135, 6319, 6036, 6327	41.8 ± 4.5	4/6	24.0 ± 0.8	24.3 ± 4.0	17.5 ± 2.2	0.0 ± 0.0	30.9 ± 1.65
T2D	6142, 6028, 6127, 6249, 6277, 6255	42.6 ± 3.9	3/3	31.0 ± 0.8	30.6 ± 5.3	12.0 ± 1.7	5.0 ± 3.5	68.4 ± 8.26
ND (adults)	6251, 6425, 6015, 6250, 6019, 6444, 6009, 6165, 6295, 6288	42.9 ± 1.9	5/5	30.1 ± 1.1			6.6 ± 1.4	87.2 ± 7.68

Data are presented as means ± SEM

Gender was reported prior to organ recovery procedures

ND, non-diabetic; T1D, type 1 diabetes; T2D, type 2 diabetes

### Immunofluorescence staining and image acquisition

After validating the PC1/3 antibody via immunostaining using pancreatic sections from wild-type and beta cell-specific *Pcsk1*-knockout mice (ESM Fig. 1), immunostaining of two consecutive pancreatic sections per donor was performed (a total of 104 sections were stained). A previously reported protocol [20] was used, with the following primary antibody cocktails reconstituted in an antibody diluent (S080983-2; Agilent Technologies, CA, USA): (1) insulin (IR002, Agilent Technologies, RRID:AB_2800361, pan-insulin antibody, 1:4 dilution), proinsulin (GS*-*9A8*,* Developmental Studies Hybridoma Bank, IA, USA, RRID:AB_532383, detects the B-C junction of proinsulin, 1:50 dilution), PC1/3 (ab191452, Abcam, Cambridge, UK, RRID:AB_3064864, 1:500 dilution, likely detects both 87 kDa and 66 kDa forms of PC1/3); and (2) glucagon (ab92517, Abcam, RRID:AB_10561971, 1:1000 dilution), somatostatin (NB100–64650, Novus Biologicals, CO, USA, RRID:AB_965456, 1:200 dilution) and PC1/3 (ab191452, Abcam, RRID:AB_3064864, 1:500 dilution). Of note, the insulin, proinsulin, and PC1/3 staining combination was not performed in pancreatic sections from donors #6347, #6087, #6212 and #6319, due to limited sample availability. A minimum of 30 islets from pancreases of non-diabetic donors and donors with type 2 diabetes were randomly selected and imaged, and all islets from pancreases of donors with short- and long-duration type 1 diabetes (consisting of more than ten cells in a cluster) were imaged using a Leica TCS SP5 confocal microscope (Leica Microsystems, Hesse, Germany). All images were acquired under blinded fashion using the same exposure setting across experiments and pixel intensity was kept within the dynamic range.

### Image and data analysis

**Fig. 1:**
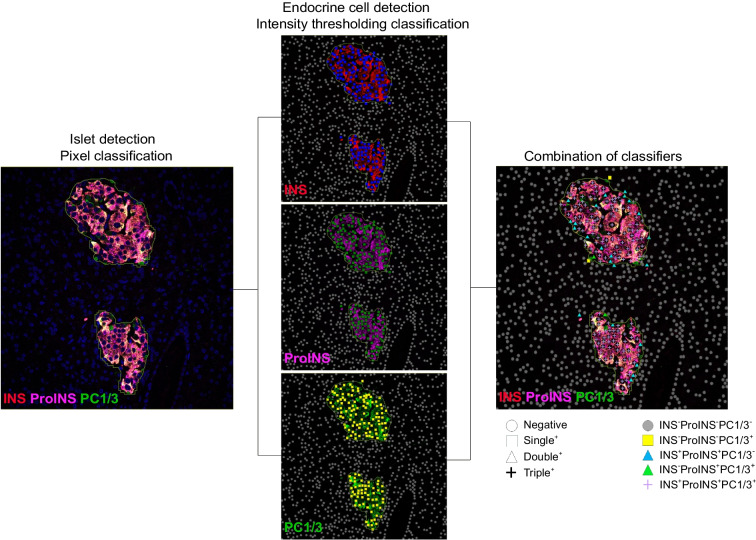
Schematic illustration of the confocal image analysis workflow using QuPath, version 0.4.3. Islets were detected using a pixel classifier. After islet detection, cells were identified using a StarDist plug-in. The single measurement classifier tool was employed to detect positive cells for the marker of interest. Cells were identified as areas of staining above the background level by applying optimised cell mean intensity thresholds. Single measurement classifiers were combined to identify negative, single-positive, double-positive or triple-positive cells and a colour code was used to differentiate different beta and alpha cell populations. INS, insulin; ProINS, proinsulin

Quantitative analysis of individual islet cells was performed using QuPath (version 0.4.3), an open-source software designed for digital pathology [21]. The extension tool StarDist, a deep-learning-based method of 2D and 3D images, was implemented on QuPath for individual cell segmentation [22]. We analysed 1572 islets stained with insulin/proinsulin/PC1/3 antibodies and 4299 islets stained with glucagon/somatostatin/PC1/3 antibodies. A total of 85,245 beta cells and 24,711 alpha cells were imaged and analysed. Image analysis workflow to identify beta, alpha and delta cells using QuPath is depicted in Fig. 1. The classification tool was first used to identify islets via a machine-learning approach, which considers the fluorescence intensity and morphological features of the following cells: (1) insulin-, proinsulin- and PC1/3-positive cells; and (2) glucagon-, somatostatin- and PC1/3-positive cells. As described previously, individual cells were segmented through localising individual cell nuclei via a StarDist plug-in using a custom script [23]. Using thresholding of the cell mean intensity signal, cells positive for insulin, proinsulin, glucagon, somatostatin and PC1/3 were compiled to define islet cell subpopulations (representative images with cell segmentation are provided in ESM Fig. 2). Finally, a master script was developed for automated islet and cell detection and classification. Minimal manual corrections were applied, if necessary. The number of cells positive for one, two or three protein markers (insulin, proinsulin, glucagon, somatostatin and PC1/3), and their staining intensity values, were obtained for further statistical analysis.

Quantitative analysis of whole islets was performed using the open-source software CellProfiler (version 4) [24]. For this step, CellProfiler was chosen instead of QuPath, because image analysis at the whole-islet level does not require advanced cell segmentation and can be performed efficiently using custom CellProfiler pipelines. In brief, after image processing (alignment, illumination correction and thresholding), islets were identified based on global thresholding using the Otsu approach [25]. Identified islets were converted from objects to images, and the islet mask images were used to subtract background signals. Islet insulin, proinsulin, PC1/3, glucagon and somatostatin staining intensity and occupied areas within the region of interest were measured. A total of 1104 images stained with insulin/proinsulin/PC1/3 antibodies and 2519 images stained with glucagon/somatostatin/PC1/3 antibodies were analysed.

### Statistical analysis

Statistical analysis was performed with one-way ANOVA (for data with Gaussian distribution) or Kruskal–Wallis (for data with non-Gaussian distribution), and mixed-effects analysis using GraphPad Prism (version 10.2.3, CA, USA). Normally distributed data are expressed as mean ± SEM; data that are not normally distributed are expressed as median (IQR).

## Results

### Reduced islet and individual alpha and beta cell area points to cell atrophy and dysfunction in type 1 diabetes

**Fig. 2:**
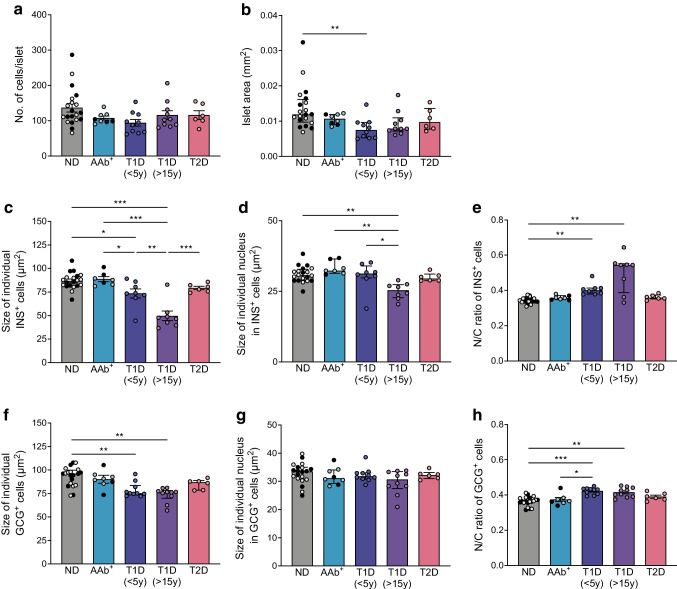
Morphological analysis of alpha and beta cells. Mean number of cells per islet (**a**), median islet area (**b**), mean size of individual insulin-positive cells (**c**), median size of individual nucleus of insulin-positive cells (**d**), median nucleus/cytoplasm ratio of insulin-positive cells (**e**), median size of individual glucagon-positive cells (**f**), mean size of individual nucleus of glucagon-positive cells (**g**) and mean nucleus/cytoplasm ratio of glucagon-positive cells (**h**) were analysed in pancreatic sections from donors. Data are shown for pancreases from non-diabetic donors (filled circles, older adult; empty circles, young adult), AAb^+^ donors (filled circles, double AAb^+^; empty circles, single AAb^+^), donors with recent-onset type 1 diabetes (duration <5 years), donors with long-duration type 1 diabetes (>15 years) and donors with type 2 diabetes. Each data point represents the average value from one donor. Normally distributed data (**a**, **c**, **g**, **h**) are expressed as mean ± SEM, non-normal data (**b**, **d**, **e**, **f**) are expressed as median ± IQR, *p<0.05, **p<0.01 and ***p<0.001. GCG, glucagon; INS, insulin; N/C, nucleus/cytoplasm; ND, non-diabetic; T1D, type 1 diabetes; T2D, type 2 diabetes; y, years

Donors with type 1 diabetes have reduced pancreas and beta cell mass. However, whether their beta cell size is reduced remains unknown. Using high-resolution islet image analysis, we showed that the mean number of cells per islet was comparable in different donor groups (Fig. 2a) while median islet area was reduced in donors with type 1 diabetes (0.012 mm^2^ vs 0.008 mm^2^ for non-diabetic vs short-duration type 1 diabetes, respectively) (Fig. 2b). We further analysed the cytoplasmic and nuclear area of individual beta and alpha cells in different donor groups. Of note, only nucleated cells were selected for cytoplasmic area analysis. The mean cytoplasmic area of beta cells positive for insulin was reduced in islets from donors with short- or long-duration type 1 diabetes (86.83 ± 1.96 μm^2^ vs 73.52 ± 4.86 μm^2^ vs 49.75 ± 5.08 μm^2^ for non-diabetic donors vs donors with short-duration type 1 diabetes vs long-duration type 1 diabetes, respectively) (Fig. 2c). The median nuclear area of beta cells was also reduced in islets from donors with long-duration type 1 diabetes (30.69 μm^2^ vs 25.40 μm^2^ for non-diabetic donors vs donors with long-duration type 1 diabetes) (Fig. 2d), whereas the median nucleus/cytoplasm ratio was elevated in beta cells from type 1 diabetes donors (0.338 vs 0.401 vs 0.544 for non-diabetic donors vs donors with short-duration type 1 diabetes vs donors with long-duration type 1 diabetes, respectively) (Fig. 2e), pointing to a more acute reduction of the cytoplasmic compared with the nuclear area in type 1 diabetes. Regarding individual alpha cells, we observed morphological changes affecting mainly the cytoplasm. The median cytoplasmic area was reduced in glucagon-positive cells (96.07 μm^2^ vs 75.15 μm^2^ vs 76.37 μm^2^ for non-diabetic donors vs donors with short-duration type 1 diabetes vs donors with long-duration type 1 diabetes, respectively) (Fig. 2f) while the nuclear area remained comparable in all donor groups (Fig. 2g). As a result of the size changes, the mean nucleus/cytoplasm ratio was increased in donors with type 1 diabetes (0.364 ± 0.006 vs 0.423 ± 0.006 vs 0.416 ± 0.009 for non-diabetic donors vs donors with short-duration type 1 diabetes vs donors with long-duration type 1 diabetes, respectively) (Fig. 2h). This indicates that both beta and alpha cells undergo morphological changes as type 1 diabetes progresses and that the decrease in cell size may correspond to a decrease in cell function. We further showed that not only endocrine cells but also islet-adjacent exocrine cells were smaller in donors with short-duration type 1 diabetes (95.76 ± 2.22 μm^2^ vs 86.42 ± 1.78 μm^2^ for non-diabetic donors vs donors with short-duration type 1 diabetes) and that pancreas weight was reduced in donors with short- or long-duration type 1 diabetes (79.82 ± 5.26 g vs 45.92 ± 6.24 g vs 30.62 ± 1.65 g for non-diabetic donors vs donors with short-duration type 1 diabetes vs donors with long-duration type 1 diabetes, respectively) (ESM Fig. 3).

### Islets from donors with type 1 diabetes have a reduced percentage of PC1/3-positive and insulin-positive cells

**Fig. 3:**
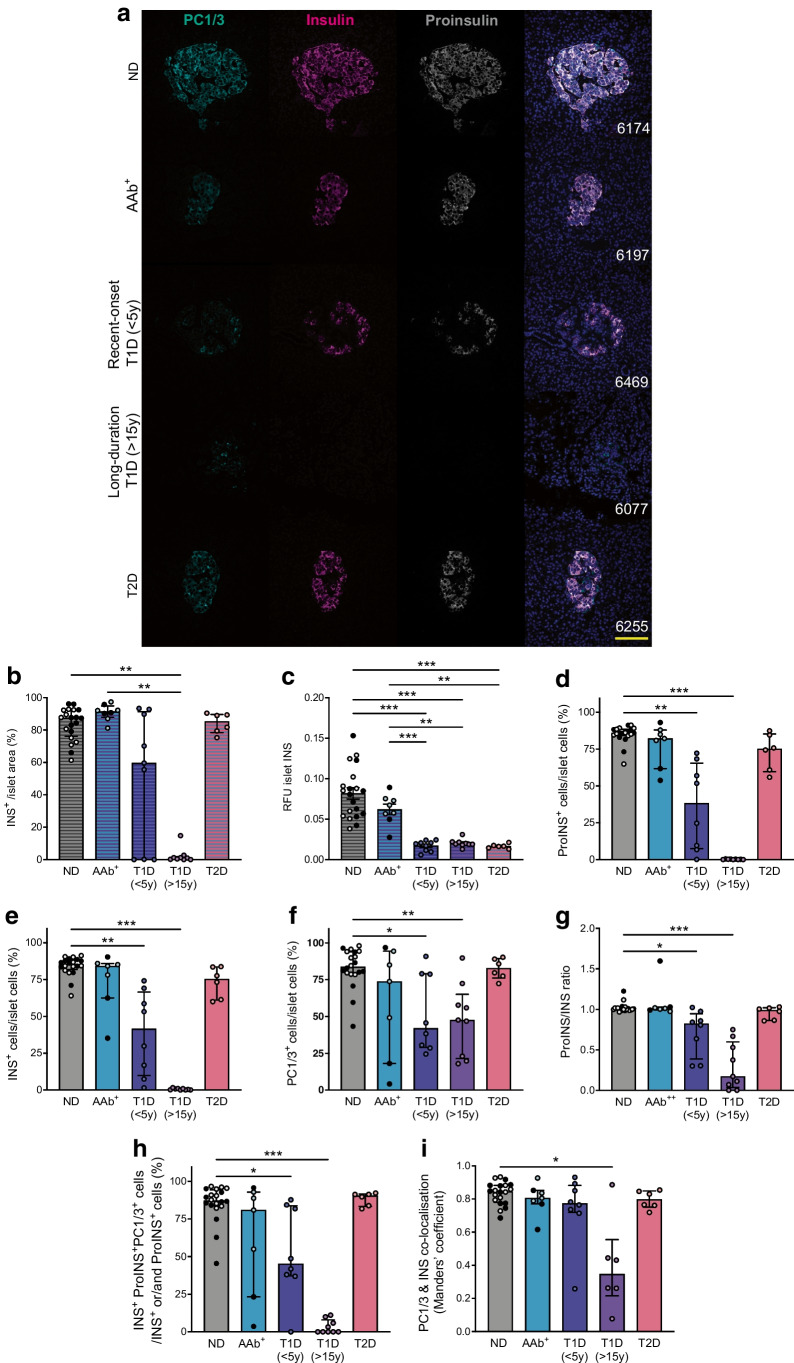
PC1/3 expression in beta cells. (**a**) Representative images showing PC1/3, insulin and proinsulin expression in islets from donors with or without diabetes. nPOD case numbers are indicated in the merged images. Scale bar, 100 μm. (**b**) Proportional insulin-positive area in islets. (**c**) RFU of insulin in donor islets. (**d**, **e**, **f**) Percentage of proinsulin- (**d**), insulin- (**e**) and PC1/3- (**f**) positive cells in donor islets. (**g**) Proinsulin/insulin RFU ratio in individual beta cells in islets from donors with or without diabetes. (**h**, **i**) Percentage of proinsulin, insulin and PC1/3 triple-positive cells (**h**) and PC1/3 and insulin co-localisation (**i**) in beta cells from donors with or without diabetes. Data are shown for islets from non-diabetic donors (filled circles, older adults; empty circles, young adults), AAb^+^ donors (filled circles, double AAb^+^; empty circles, single AAb^+^), donors with recent-onset type 1 diabetes (duration <5 years), donors with long-duration type 1 diabetes (>15 years) and donors with type 2 diabetes. Each data point represents the average value from one donor. Hatched bars represent data from images analysed at the islet level; non-hatched bars represent data from image analysed at the single-cell level. Normally distributed data (**c**) are expressed as mean ± SEM, non-normal data (**b**, **d**–**i**) are expressed as median ± IQR, *p<0.05, **p<0.01 and ***p<0.001. INS, insulin; ND, non-diabetic; ProINS, proinsulin; T1D, type 1 diabetes; T2D, type 2 diabetes; y, years

In agreement with previous reports, our islet-level image analysis (using CellProfiler software) showed that the median insulin-positive area was decreased in donors with long-duration type 1 diabetes (87.52% vs 0.97% for non-diabetic donors vs donors with long-duration type 1 diabetes, respectively) (Fig. 3a, b). Mean fluorescence staining intensity of insulin was also reduced in donors with type 1 diabetes (0.082 ± 0.007 relative fluorescence units [RFU] vs 0.017 ± 0.002 RFU vs 0.020 ± 0.002 RFU for non-diabetic donors vs donors with short-duration type 1 diabetes vs donors with long-duration type 1 diabetes) (Fig. 3c). These findings were recapitulated via single-cell image analysis using QuPath. We showed that the percentages of insulin-positive cells and proinsulin-positive cells were significantly reduced in islets from donors with recent-onset or long-duration type 1 diabetes (median percentage of proinsulin-positive cells in islets was 85.94% vs 38.25% vs 0.19% for non-diabetic donors vs donors with short-duration type 1 diabetes vs donors with long-duration type 1 diabetes, respectively; the respective median percentage of insulin-positive cells in islet was 85.34% vs 41.55% vs 0.41%) (Fig. 3d, e). The median percentage of PC1/3-positive cells was also reduced in islets from donors with type 1 diabetes (83.75% vs 42.01% vs 47.64% for non-diabetic donors vs donors with short-duration type 1 diabetes vs long-duration type 1 diabetes, respectively). More than 45% of islet cells remained PC1/3-positive in islets from donors with long-duration type 1 diabetes (Fig. 3f). The median proinsulin/insulin ratio was significantly reduced in islets from donors with type 1 diabetes (1.009 vs 0.823 vs 0.171 for non-diabetic donors vs donors with short-duration type 1 diabetes vs donors with long-duration type 1 diabetes) (Fig. 3g). As expected, the median percentage of insulin-, proinsulin- and PC1/3-positive cells in islets decreased substantially in donors with type 1 diabetes (87.23% vs 45.26% vs 0.00%, non-diabetic vs short-duration type 1 diabetes vs long-duration type 1 diabetes) (Fig. 3h). Furthermore, islet insulin and PC1/3 co-localisation was significantly reduced in donors with long-duration type 1 diabetes (0.845 vs 0.347 for non-diabetic donors vs donors with long-duration type 1 diabetes, respectively) (Fig. 3i), suggesting reduced prohormone-processing capacity in residual beta cells.

**Fig. 4:**
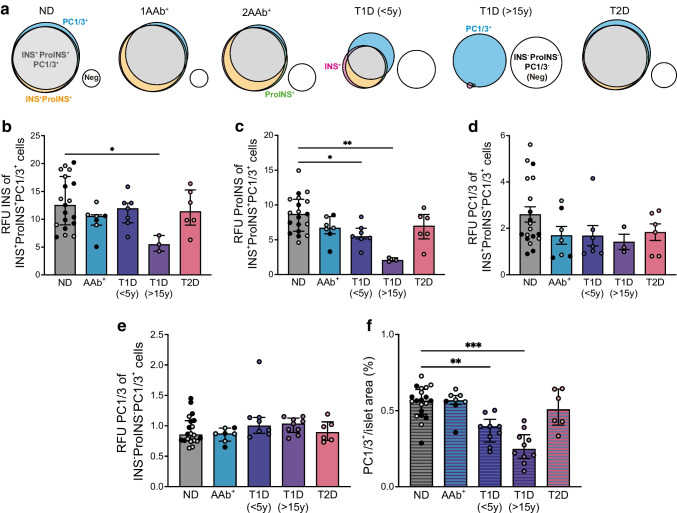
Trajectory of islet PC1/3 expression in diabetes. (**a**) Venn diagram showing the dynamic of cells positive for insulin (pink circle), proinsulin (green circle), PC1/3 (blue circle), both insulin and proinsulin (orange circle), or positive (grey circles) or negative (white circles) for insulin, proinsulin and PC1/3, in the progression of type 1 diabetes. Non-diabetic donors, *n*=20; single AAb^+^ donors (1AAb^+^), *n*=4; double AAb^+^ donors (2AAb^+^), *n*=3; donors with recent-onset type 1 diabetes (duration <5 years), *n*=8; donors with long-duration type 1 diabetes (>15 years), *n*=8; donors with type 2 diabetes, *n*=6. (**b**–**d**) RFU of insulin (**b**), proinsulin (**c**) and PC1/3 (**d**) in insulin-, proinsulin- and PC1/3-positive cells in donor islets. (**e**) RFU of PC1/3 in insulin-negative, proinsulin-negative and PC1/3-positive cells from donors with or without diabetes. (**f**) Percentage of PC1/3-positive area in islets from donors with or without diabetes. Data are shown for islets from non-diabetic donors (filled circles, older adults; empty circles, young adults), AAb^+^ donors (filled circles, double AAb^+^; empty circles, single AAb^+^), donors with recent-onset type 1 diabetes, donors with long-duration type 1 diabetes and donors with type 2 diabetes. The average read-out of all cells from each donor was represented as one data point. Hatched bars indicate data from images analysed at the islet level; non-hatched bars represent images analysed at the single-cell level. Normally distributed data (**d**) are expressed as mean ± SEM, non-normal data (**b**, **c**, **e**, **f**) are expressed as median ± IQR, *p<0.05, **p<0.01 and ***p<0.001. INS, insulin; ND, non-diabetic; ProINS, proinsulin; T1D, type 1 diabetes; T2D, type 2 diabetes; y, years

To gain a holistic view over different cell populations, we generated a Venn diagram, to reveal the gradual shift of islet cells from triple-positive states to either single PC1/3-positive, or triple-negative states during the progression toward long-standing type 1 diabetes (Fig. 4a). Median relative fluorescence intensity of insulin and proinsulin were reduced in the triple-positive cells of donors with type 1 diabetes (of insulin, 12.55 vs 5.47 RFU for cells from non-diabetic donors vs donors with long-duration type 1 diabetes, respectively; of proinsulin, 8.69 vs 5.48 vs 2.05 RFU for non-diabetic donors vs donors with short-duration type 1 diabetes vs long-duration type 1 diabetes, respectively) (Fig. 4b, c), hinting at steady loss of beta cell insulin granules. PC1/3 fluorescence intensity, however, was not significantly reduced in the triple-positive cells and insulin-negative cells from donors with type 1 diabetes (Fig. 4d, e). Collectively, our data suggested that it is not the expression of PC1/3 in insulin-containing beta cells (Fig. 4d) but the co-localisation of PC1/3 and insulin at a subcellular level (Fig. 3i) that is significantly diminished in donors with type 1 diabetes. This suggests that defective protein trafficking may be a key contributor to insufficient beta cell prohormone processing. At the whole-islet level, reduced PC1/3 expression (56.49% vs 39.60% vs 24.81%, non-diabetic vs short-duration type 1 diabetes vs long-duration type 1 diabetes) (Fig. 4f) likely resulted from the increase in the triple-negative cell population.

### Alpha cells expressing PC1/3 have higher levels of glucagon and increased co-localisation of PC1/3 and glucagon

**Fig. 5:**
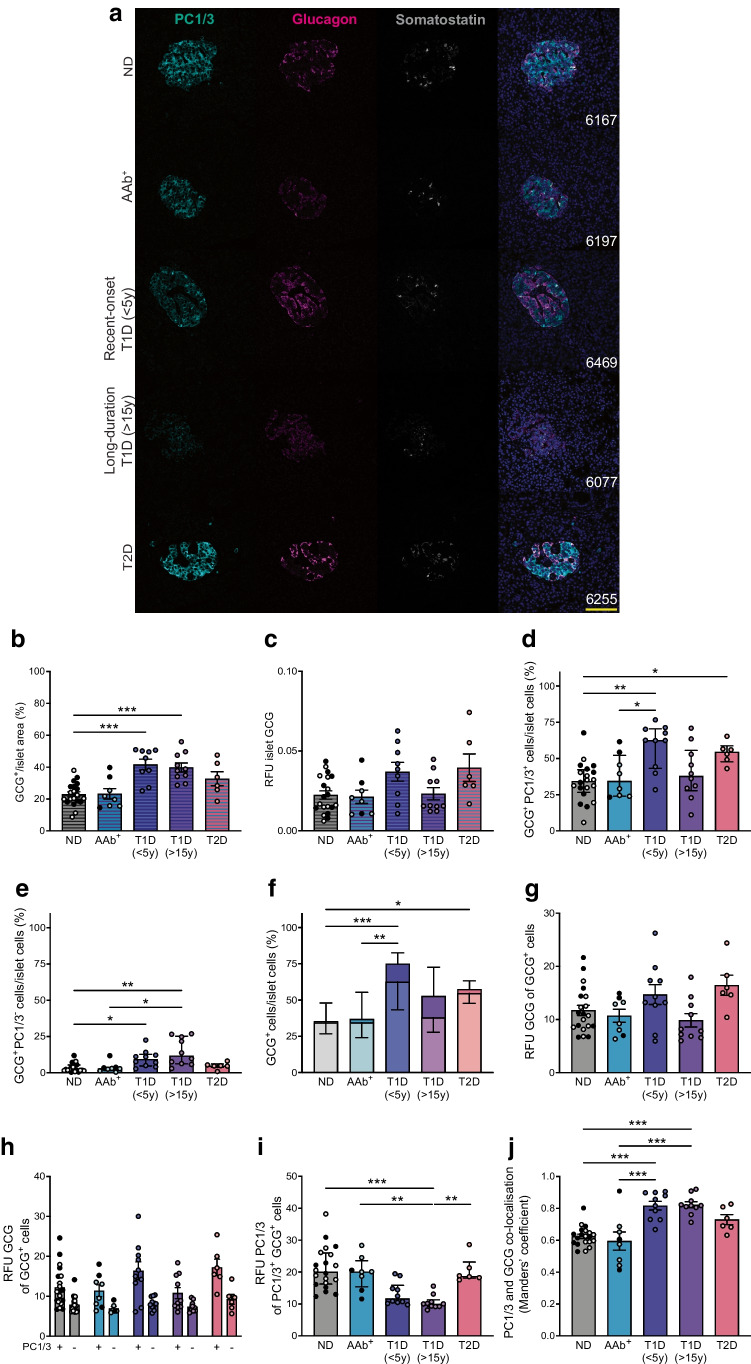
PC1/3 expression in alpha cells. (**a**) Representative images showing PC1/3, glucagon and somatostatin expression in islets from donors with or without diabetes. nPOD case numbers are indicated in the merged images. Scale bar, 100 μm. (**b**) Proportional glucagon-positive area in islets. (**c**) Median RFU of glucagon in islets. (**d**–**f**) Percentage of glucagon-positive, PC1/3-positive (**d**), glucagon-positive, PC1/3-negative (**e**) and glucagon-positive islet cells (**f**) in islets. In (**f**) the darker columns represent PC1/3-negative cells, the lighter columns represent PC1/3-positive cells. (**g**) Mean RFU of glucagon in glucagon-positive alpha cells. (**h**) Mean RFU of glucagon in PC1/3-positive vs PC1/3-negative cells. (**i**) Median RFU of PC1/3 in PC1/3-positive, glucagon-positive alpha cells. (**j**) Mean PC1/3 and glucagon co-localisation in islets. Data are shown for islets from non-diabetic donors (filled circles, older adults; empty circles, young adults), AAb^+^ donors (filled circles, double AAb^+^; empty circles, single AAb^+^), donors with recent-onset type 1 diabetes (duration <5 years), donors with long-duration type 1 diabetes (>15 years) and donors with type 2 diabetes. Each data point represents the average value from one donor. Hatched bars show data for images analysed at the islet level; non-hatched bars show data for images analysed at the single-cell level. Normally distributed data (**b**, **g**, **h**, **j**) are expressed as mean ± SEM, non-normal data (**c**, **d**, **e**, **f**, **i**) are expressed as median ± IQR. *p<0.05, **p<0.01 and ***p<0.001. GCG, glucagon; ND, non-diabetic; T1D, type 1 diabetes; T2D, type 2 diabetes; y, years

PC1/3 is expressed in human alpha cells and may contribute to elevated GLP-1 secretion in islets from donors with type 2 diabetes [26]. Whether PC1/3 expression is elevated in alpha cells in islets from donors with type 1 diabetes remains unknown. We examined PC1/3 expression patterns in alpha cells in AAb^+^ donors, or donors with type 1 diabetes or type 2 diabetes (Fig. 5a). First, alpha cells were analysed at the whole-islet level via CellProfiler (Fig. 5b, c). The mean islet glucagon-positive area was increased in donors with type 1 diabetes (22.94 ± 1.61% vs 41.61 ± 3.41% vs 39.79 ± 2.81% for non-diabetic donors vs donors with short-duration type 1 diabetes vs long-duration type 1 diabetes, respectively) (Fig. 5b), yet glucagon expression in islets was highly heterogenous, and the median islet fluorescence intensity of glucagon was comparable among donor groups (Fig. 5c). Then, the median percentage of glucagon-positive, PC1/3-positive cells was calculated at the single-cell level using QuPath. Alpha cells expressing PC1/3 were increased in donors with short-duration type 1 diabetes, as well as donors with type 2 diabetes (34.30% vs 62.53% vs 54.54% for non-diabetic donors vs donors with short-duration type 1 diabetes vs type 2 diabetes, respectively) (Fig. 5d). Glucagon-positive, PC1/3-negative cells were also increased in islets from donors with type 1 diabetes (2.04% vs 9.29% vs 11.73% for non-diabetic donors vs donors with short-duration type 1 diabetes vs long-duration type 1 diabetes, respectively) (Fig. 5e). In aggregate, the percentage of glucagon-positive cells (the sum of PC1/3-positive and PC1/3-negative alpha cells), was significantly increased in donors with both type 1 diabetes and type 2 diabetes (35.52% vs 75.11% vs 57.53 for non-diabetic donors vs donors with short-duration type 1 diabetes vs type 2 diabetes, respectively) (Fig. 5f). Glucagon expression on a cellular basis, evaluated by mean fluorescence intensity, remained comparable among all donor groups (Fig. 5g). We also compared the expression of glucagon in PC1/3-positive, glucagon-positive cells and PC1/3-negative, glucagon-positive cells and found that PC1/3-expressing cells had significantly higher glucagon staining intensity (mixed-effects analysis, *p*<0.05 for PC1/3-positive cells vs PC1/3-negative cells, Fig. 5h). PC1/3 median staining intensity was reduced in glucagon-positive cells in donors with type 1 diabetes (20.09% vs 10.03% for non-diabetic donors vs donors with long-duration type 1 diabetes, respectively) (Fig. 5i), yet its co-localisation with glucagon at the single-cell level was increased in donors with type 1 diabetes (Manders’ coefficient: 0.624 ± 0.014 vs 0.817 ± 0.027 vs 0.730 ± 0.313 for non-diabetic donors vs donors with short-duration type 1 diabetes vs long-duration type 1 diabetes, respectively) (Fig. 5j), hinting toward potentially enhanced glucagon processing via PC1/3 in type 1 diabetes.

### Islet delta cell area is increased in donors with type 1 diabetes

**Fig. 6:**
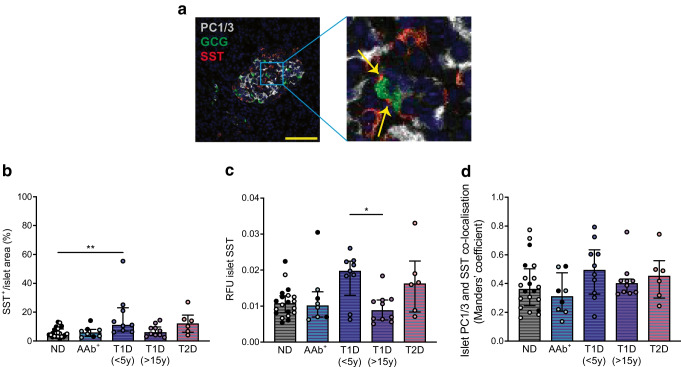
Glucagon and somatostatin expression pattern in islets. (**a**) Representative images showing PC1/3, glucagon and somatostatin expressions in islets from a donor without diabetes. Yellow arrows indicate the cytoplasmic processes of delta cells. Scale bar, 100 μm. (**b**–**d**) Proportional somatostatin-positive area (**b**), median RFU of somatostatin (**c**) and co-localisation of PC1/3 and somatostatin (**d**) in islets from non-diabetic donors (filled circles, older adults; empty circles, young adults), AAb^+^ donors (filled circled, double AAb^+^; empty circles, single AAb^+^), donors with recent-onset type 1 diabetes (duration <5 years), donors with long-duration type 1 diabetes (>15 years) and donors with type 2 diabetes. Each data point represents the average value from one donor. Data are expressed as median ± IQR. *p<0.05 and **p<0.01. GCG, glucagon; ND, non-diabetic; SST, somatostatin; T1D, type 1 diabetes; T2D, type 2 diabetes; y, years

Expression of PC1/3 in somatostatin-positive delta cells, unfortunately, could not be quantified at the single-cell level due to limitations in cell segmentation. Delta cells have elongated cell profiles [27] that wrap around other islet cells, which makes quantification of delta cell number challenging and inaccurate on a 2D setting in thin tissue sections (Fig. 6a) Therefore, delta cells were analysed at the whole-islet level only using CellProfiler. Interestingly, the median somatostatin-positive area was significantly elevated in islets from donors with short-duration type 1 diabetes (4.68% vs 11.12% for non-diabetic donors vs donors with short-duration type 1 diabetes, respectively) (Fig. 6b). Islet somatostatin expression levels also varied among donors, and the median fluorescence intensity of somatostatin in islets was comparable between non-diabetic donors and donors with type 1 diabetes (Fig. 6c). Co-localisation analysis of islet PC1/3 and somatostatin showed no significant differences among donor groups (Fig. 6d).

## Discussion

Elevated plasma proinsulin/C-peptide and proIAPP/IAPP ratios are promising biomarkers of beta cell dysfunction, can potentially be used to predict the onset of type 1 diabetes, islet graft failure, and response to immunotherapy [8, 12, 20, 28], and may be associated with loss of PC1/3 expression in islets [9–11]. Considering that PC1/3 is expressed in all major islet cell types, further analysis of PC1/3 expression at single-cell resolution in donors with or without diabetes is needed to provide insight into endocrine cell dysfunction and adaptation during the pathogenesis of diabetes. To this end, we quantitatively analysed the expression of proinsulin, insulin, PC1/3, glucagon and somatostatin at the islet and single-cell level, using the open-source digital pathology analysis software programs CellProfiler and QuPath, on pancreatic sections from 54 organ donors with or without diabetes.

We showed that the size of both beta and alpha cells is reduced in donors with type 1 diabetes. Moreover, average beta cell size further declined as type 1 diabetes progressed, suggesting that pathophysiological factors continue to sculpt the morphology of beta cells after the onset of type 1 diabetes. The scope of pancreatic cell atrophy extends beyond endocrine lineage, as we found that exocrine cell size is also reduced in donors with short-duration type 1 diabetes, and pancreas weight is reduced in donors with short- or long-duration type 1 diabetes (ESM Fig. 3) [29, 30]. As previously reported [31], we also observed progressive loss of beta cell granule peptide hormone content. While the percentage of insulin, proinsulin and PC1/3 triple-positive cells in islets is significantly reduced in donors with type 1 diabetes (Fig. 4a), the expression levels of insulin and proinsulin in the remaining beta cell population further declines in donors with long-duration type 1 diabetes. Thus, it may be of therapeutic value to preserve beta cells across all clinical stages of type 1 diabetes progression, to mitigate the deterioration of beta cell morphology (i.e. size) and identity (i.e. insulin and PC1/3 co-localisation, and insulin content).

In comparison with previous findings demonstrating elevated proinsulin/insulin ratio in plasma of adults with established type 1 diabetes [32] and increased proinsulin/insulin area ratio in whole-slide pancreas sections from donors with recently-diagnosed type 1 diabetes [33], we found that the proinsulin/total insulin intensity ratio is not increased and that proinsulin-positive cells and insulin-positive cells are both significantly reduced in islets from donors with type 1 diabetes. This discrepancy in the prohormone/hormone ratio in plasma vs islets could be due to a release and depletion of cellular proinsulin stores in type 1 diabetes. In addition, donors in the current study are older, while pancreatic samples analysed in the previous study were collected from living individuals within weeks of type 1 diabetes diagnosis. It is plausible that as type 1 diabetes progresses, depletion of insulin granules and overall beta cell loss leads to reduced islet proinsulin expression. We infer that PC1/3-mediated prohormone processing is impaired in beta cells in donors with type 1 diabetes, as the percentage of PC1/3-positive and insulin-positive cells in islets is reduced, and the co-localisation of PC1/3 and insulin at the subcellular level is significantly decreased. Unfortunately, due to the complex aetiology of type 1 diabetes and limited numbers of AAb^+^ donors analysed, we were not able to determine whether PC1/3-mediated processing is altered in islet cells prior to the onset of type 1 diabetes.

Although the pathogenesis of type 1 diabetes and type 2 diabetes is fundamentally distinct [34], islet cell adaptation mechanisms are similar [35]. Canonically, proglucagon is processed via PC2 into bioactive glucagon in alpha cells. Under experimentally induced islet stress, proglucagon may be processed by PC1/3 into GLP-1 to promote insulin secretion and beta cell survival [36]. Our results, together with previous findings demonstrating elevated islet alpha cell *PCSK1* transcript and GLP-1 expression in islets from donors with type 2 diabetes [14, 37], point towards alpha cell adaptation. We further showed that the percentage of glucagon and PC1/3 double-positive cells is also elevated in islets from donors with recent-onset type 1 diabetes. Additional analyses of amidated GLP-1 in alpha cells and co-expression of insulin and glucagon are needed to support the hypothesis that alpha cells adapt in type 1 diabetes by acquiring beta cell identity and produce GLP-1 in islets. Because PC1/3 and PC2 could play compensatory roles in prohormone processing, and it has been reported that PC2 expression in beta cells is elevated in donors with type 2 diabetes [38], further analysis of PC2 expression in islet cells could provide comprehensive insights into adaptive peptide hormone processing in type 1 diabetes.

Recent morphological studies have shown that delta cells have cytoplasmic processes extending up to 22 μm (one-third of the islet diameter), making in situ single-cell analysis (especially cell segmentation) challenging. Therefore, we analysed the somatostatin-positive area in islets and found that both delta cell area and somatostatin staining intensity are increased in donors with recent-onset type 1 diabetes. Our results agree with previous reports describing delta cell mass expansion in rodents and in donors with type 1 diabetes [39–41]. Of note, the increment in delta cell area in islets from donors with type 2 diabetes did not reach statistical significance in our study, and further histopathological studies using 3D islet imaging are needed to fully evaluate the distribution and morphology of delta cells in humans during the development of diabetes.

There are several limitations to this study. First, we did not examine pancreatic sections from children with type 1 diabetes. Second, we manually acquired images using a confocal microscope to obtain high-resolution images, and we were only able to analyse a smaller number of islets in donors with type 1 diabetes. Cell clusters containing fewer than ten endocrine cells, and large islets exceeding the field of view were not included in our analysis. Third, the impact of gender on islet prohormone processing was not analysed, as this study was not powered to detect gender differences. Finally, only basic cellular markers were used to identify different endocrine cells. Additional multiplex immunostaining experiments to include beta cell identity and endocrine cell markers [42, 43], and islet hormone processing products (such as amidated GLP-1), could be used to unfold the fluidity of islet cell fates during development of diabetes.

In conclusion, through comprehensive and quantitative immunohistological analysis, we showed that islet beta cells undergo morphological and functional deterioration as type 1 diabetes progresses. While PC1/3-mediated proinsulin processing might be reduced, PC1/3-mediated proglucagon processing seems to increase, pointing towards potential alpha cell adaptation and compensation during type 1 diabetes. Our findings suggest that fostering beta cell preservation and islet cell adaptation, both before and after the diagnosis of type 1 diabetes, may be of therapeutic importance.

## Supplementary Information

Below is the link to the electronic supplementary material.

ESM Figs (PDF 28.7 MB)

## Data Availability

Raw data and images are available upon request.

## Abbreviations

AAb^+^: Autoantibody-positive
CPE: Carboxypeptidase E
GLP-1: Glucagon-like peptide 1
IAPP: Islet amyloid polypeptide
nPOD: Network for Pancreatic Organ Donors with Diabetes
PC1/3: Prohormone convertase 1/3
PC2: Prohormone convertase 2
proIAPP: Pro-islet amyloid polypeptide
RFU: Relative fluorescence units
