# Supplementary material for: Quantitative analysis of islet prohormone convertase 1/3 expression in human pancreas donors with diabetes
Source: Diabetologia. 2024 Oct 15;67(12):2771–85. doi: 10.1007/s00125-024-06275-5 (PMC11604696; doi:10.1007/s00125-024-06275-5)
Supplement: Supplementary file 1 — ESM Figs (PDF 28.7 MB) [file 125_2024_6275_MOESM1_ESM.pdf]

a

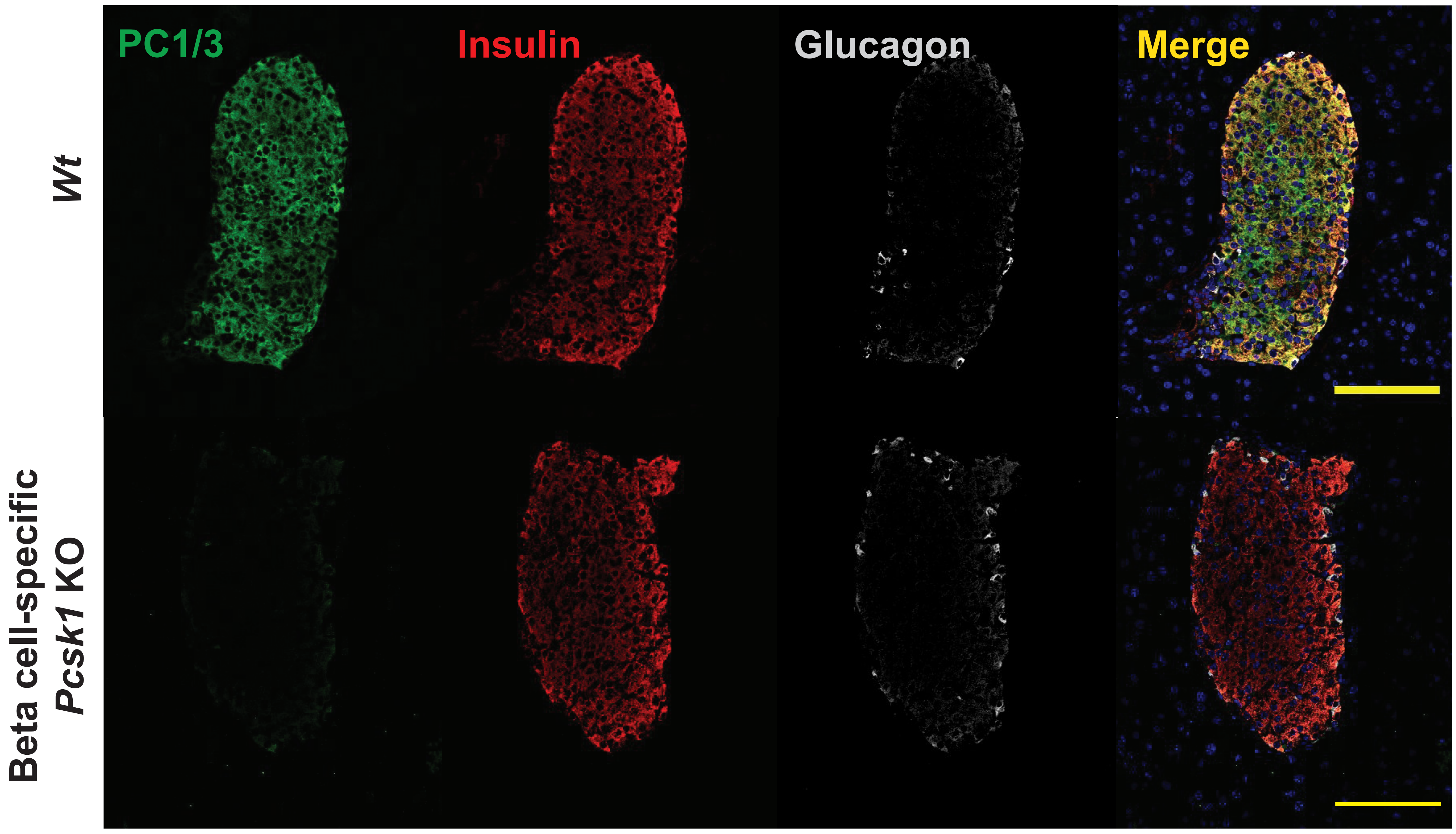

b

PC1/3 antibody targeting region

```
|NEC1_HUMAN       DVFYNT---KPYKHRDDRLLQALVDILNEEN----------------------------- 753
|NEC2_HUMAN       ------------------------------------------------------------ 638
|PCSK7_HUMAN      ----------------------------------------C------------------- 785
|PCSK9_HUMAN      ------------------------------------------------------------ 692
|FURIN_HUMAN      TVFLVLQLRSGFSFRGVKVYTMDRGLISYKGLPPEAWQEECPSDSEEDEGRGERTAFIKD 790
```

ESM. Figure 1 (a) Representative images showing PC1/3, insulin, and glucagon staining in islets from wild-type (*Wt*) and beta cell-specific Pcsk1 knockout (KO) mice. **(b)** PC1/3 antibody-targeted sequence. NEC1, neuroendocrine convertase PC1, PC1/3; NEC2, neuroendocrine convertase PC2, PC2.

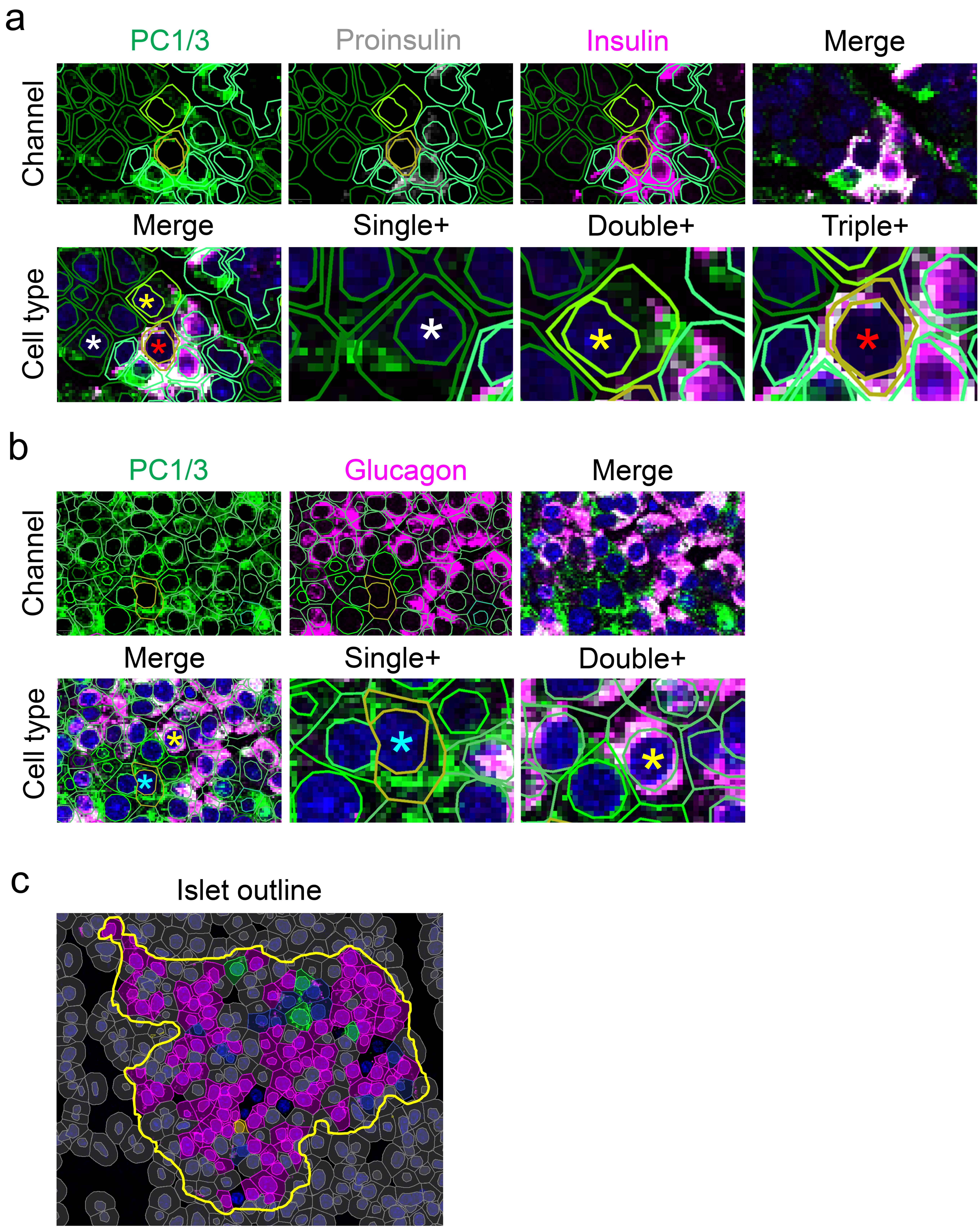

ESM. Figure 2 Representative images showing cell segmentation based on **(a)** PC1/3, proinsulin, and insulin or **(b)** PC1/3 and glucagon staining signals, using QuPath software with StarDist plug-in. Asterisks indicate cells with single-, double-, or triple- markers. **(c)** Representative image showing islet ouline generated via advanced cell detection and classification pipelines in QuPath.

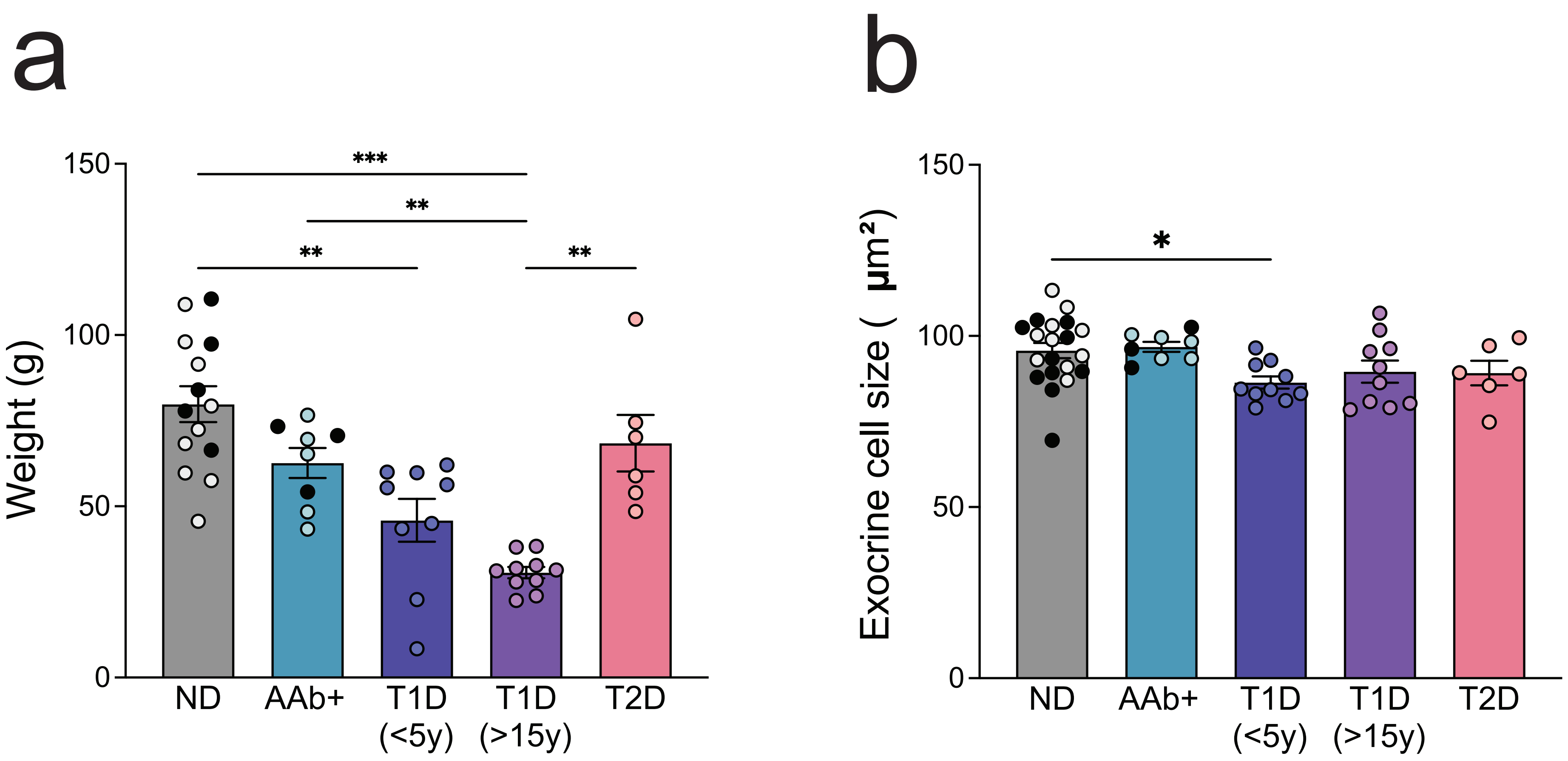

ESM. Figure 3 (a) Average pancreas weight of non-diabetic donors (ND; filled circle: older adult; empty circle, young adult, n=5 and 9), donors with autoantibodies (AAb+; filled circle: double autoantibody-positive, n=3; empty circle: single autoantibody-positive, n=5), donors with recent-onset T1D (T1D <5y, n=9), long-duration T1D (T1D >15y, n=10) and donors with T2D (n=6). **(b)** Average exocrine cell size in pancreas of non-diabetic donors (ND; filled circle: older adult; empty circle, young adult, n=10 each group), donors with autoantibodies (AAb+; filled circle: double autoantibody-positive, n=3; empty circle: single autoantibody-positive, n=5), donors with recent-onset T1D (T1D <5y, n=10), long-duration T1D (T1D >15y, n=10) and donors with T2D (n=6). Each dot represents the average value from one donor, data are expressed as mean ± SEM, *p<0.05, ** p<0.01, ***p<0.001.
